# Network analysis of gene fusions in human cancer

**DOI:** 10.1186/1471-2105-14-S17-A13

**Published:** 2013-10-22

**Authors:** Morgan Harrell, Junfeng Xia, Zhongming Zhao

**Affiliations:** 1 Department of Biomedical Informatics, Vanderbilt University School of Medicine, Nashville, TN 37203, USA; 2 Department of Psychiatry, Vanderbilt University School of Medicine, Nashville, TN 37232, USA; 3 Department of Cancer Biology, Vanderbilt University Medical Center, Nashville, TN 37232, USA

## Background

Gene fusions are hybrid genes formed when two discrete genes are incorrectly joined together. Gene fusions are found to play roles in tumorigenesis. For example, the fusion gene *BCR-ABL* translates into an abnormal tyrosine kinase that accelerates development of chronic myelogenous leukemia [1]. A network is a relational representation of nodes (e.g., genes) with edges, and is a useful approach to explore biological interactions among many related nodes. Network analysis of gene fusions in cancer would aid the exploration of gene fusion occurrence and association with tumorigenesis. Hoglund et al [2] performed an initial investigation of gene fusions network after collecting 291 tumorigenesis related gene fusions from the Mitelman database in 2006. Since then, gene fusion data has exponentially increased. There is no current and comprehensive cancer-related gene fusion network to assist in targeting cancer-associated genes.

## Materials and methods

We mined three public databases for cancer-related gene fusion sequences, and one database for fusions records from cancer studies, transcriptome analysis, and genetic disorders. Specifically, we processed each dataset by removing incomplete entries and then extracted gene-fusion pairs. Genes serve as the nodes in the network and each fusion pair is joined by an edge. Repeating pairs were represented once. We used Cytoscape to build five networks: one for each dataset and the fifth that encompasses all datasets. We graphed the occurrence of degree in each single dataset network to determine an empirical definition for hub genes.

## Results

The comprehensive network includes 9852 genes, displays 12,791 relationships, and highlights 1248 hub genes. The network highlights genes such as *MLL* and *MALAT1*, both of which have roles in tumorigenesis. The network also highlights genes such as *WDR74* and *COL1A1*, which are not much studied.

## Conclusions

This preliminary network analysis provides interesting features of tumorigenesis-related fusions. Further systematic analysis of gene fusion networks may aid researchers to better understand cancer gene fusions and test novel fusions in specific types of cancer.
